# Paricalcitol Attenuates Contrast-Induced Acute Kidney Injury by Regulating Mitophagy and Senescence

**DOI:** 10.1155/2020/7627934

**Published:** 2020-11-23

**Authors:** Eunjin Bae, Jin Hyun Kim, Myeong Hee Jung, Si Jung Jang, Tae Won Lee, Sehyun Jung, Seunghye Lee, Ha Nee Jang, Se-Ho Chang, Dong Jun Park

**Affiliations:** ^1^Department of Internal Medicine, Gyeongsang National University Changwon Hospital, Changwon, Republic of Korea; ^2^Department of Internal Medicine, College of Medicine, Gyeongsang National University, Jinju, Republic of Korea; ^3^Institute of Health Science, College of Medicine, Gyeongsang National University, Jinju, Republic of Korea; ^4^Biomedical Research Institute, Gyeongsang National University Hospital, Jinju, Republic of Korea; ^5^Department of Internal Medicine, Gyeongsang National University Hospital, Jinju, Republic of Korea

## Abstract

Contrast-induced acute kidney injury (CI-AKI) is the third most common cause of hospital-acquired renal failure, with an incidence of 11%. However, the disease mechanism remains unclear, and no effective treatment is available. Paricalcitol has been reported to be effective in animal models of kidney injury. We hypothesized that paricalcitol could play a renoprotective role against CI-AKI. Rats were divided into control, paricalcitol, contrast, and paricalcitol-plus-contrast groups. We used a previously published protocol to produce CI-AKI. Paricalcitol (0.3 *μ*g/kg) was administered intraperitoneally before 24 h and 30 min before indomethacin. We used HK-2 cells to evaluate the effects of paricalcitol on mitophagy and senescence. Ioversol triggered renal dysfunction, increasing blood urea nitrogen and serum creatinine. Significant tubular damage, increased 8-OHdG expression, and apoptosis were apparent. Ioversol injection induced high expression levels of the mitophagy markers Pink1, Parkin, and LC3 and the senescence markers *β*-galactosidase and p16INK4A. Paricalcitol pretreatment prevented renal dysfunction and reduced tissue damage by reducing both mitophagy and senescence. Cellular morphological changes were found, and expression of LC3B and HMGB1 was increased by ioversol in HK-2 cells. Paricalcitol countered these effects. This study showed that mitochondria might drive injury phenotypes in CI-AKI, and that paricalcitol protects against CI-AKI by decreasing mitochondrial damage.

## 1. Introduction

Radiocontrast agents are diagnostically and therapeutically indispensable. However, the incidence of adverse events is 1–15% despite the introduction of newer and safer materials [1]. Contrast-induced acute kidney injury (CI-AKI), a severe adverse event, refers to AKI that develops after intravascular administration of contrast media (several definitions have been published). It is the third most common cause of hospital-acquired AKI and is associated with increased short- and long-term morbidity and mortality [2]. Although the pathophysiology of CI-AKI is complex and poorly understood, it usually features medullary ischemia caused by hemodynamic changes, reactive oxygen species (ROS) formation, and tubular toxicity as reflected by cell swelling, blebbing, and apoptosis [3, 4]. Mitophagy is activated in renal tubules. Mitophagy, or autophagy of the mitochondria, is important for mitochondrial quality control, and its activation exerts renoprotective effects in CI-AKI [5]. Mitophagy modulates cell apoptosis [6] and ROS removal, eliminates damaged mitochondria, relieves inflammatory responses [7], and inhibits NLRP3 inflammasome activation in AKI [8, 9]. In addition, mitochondrial dysfunction accelerates cellular senescence [10], which is defined as irreversible cell cycle arrest, and mitophagy mitigates cellular senescence [11, 12]. Autophagy and senescence are two distinct cellular responses to stress. Although the two processes have been functionally linked [13, 14], their relationship in the CI-AKI context has not been explored.

Paricalcitol, an active vitamin D analogue, is currently used for the prevention and treatment of secondary hyperparathyroidism in patients with chronic kidney disease [15]. Several experimental studies have shown that paricalcitol (19-nor-1,25-dihydroxyvitamin D2) has beneficial effects in several models of AKI; it exhibits anti-inflammatory, antiapoptotic, and antifibrotic actions [16–20]. However, this is the first study to explore whether paricalcitol protects against experimental CI-AKI in rodents.

## 2. Materials and Methods

### 2.1. Ethical Statement

Male Sprague-Dawley (SD) rats (230–250 g; Koatech Inc., Peongtaek, Korea) were maintained under a 12 h/12 h light/dark cycle in a temperature- and humidity-controlled facility. Standard mouse chow and water were provided ad libitum. All animal experiments were performed in accordance with the National Institutes of Health Guide for the Care and Use of Laboratory Animals. The study was approved by the Gyeongsang National University Institutional Animal Care and Ethics Committee (GNU-170525-R0022).

### 2.2. Animal Experiments

Twenty-eight rats were divided into four groups: control (Con, *n* = 7), paricalcitol alone (PC, *n* = 7), contrast alone (CONT, *n* = 7), and paricalcitol prior to contrast infusion (PC+CONT, *n* = 7). We used a previously published protocol to produce CI-AKI [21]. Rats were initially given indomethacin (10 mg/kg; Wako Pure Chemical Corporation, Osaka, Japan), which was followed by N-*ω* nitro-L-arginine methyl ester (10 mg/kg; Wako Pure Chemical Corporation) and ioversol (8.3 mL/kg of organically bound iodine) via intravenous injection into the tail vein 15 and 30 min later, respectively. Paricalcitol 0.3 *μ*g/kg (Abbott Co., Seoul, Korea) was given intraperitoneally 24 h and 30 min before indomethacin (Supplementary Fig. 1). Controls received phosphate-buffered saline (PBS). Rats were sacrificed 6, 12, 24, and 48 h after ioversol injection, and blood and kidney tissues were harvested.

### 2.3. Renal Function Assessment

Blood urea nitrogen (BUN) and serum creatinine (Cr) were autoanalyzed using a diagnostic kit (Bayer, Pittsburgh, PA, USA).

### 2.4. Renal Pathology

Kidneys were fixed in 4% (*v*/*v*) phosphate-buffered paraformaldehyde, paraffin-embedded, sectioned at a thickness of 5 *μ*m, and stained with periodic acid-Schiff (PAS). Staining was semiquantitatively scored in terms of tubular injury, and scores of 0 to 4 were assigned. The tubular injury scoring system was modified from previous studies [22, 23]. Tubular injury was defined as tubular epithelial necrosis, intratubular debris, and loss of the brush border, and was scored according to the percentage of affected tubules per high-power field (×400 magnification), as follows: 0, 0%; 0.5, <10%; 1, 10–25%; 2, 26–50%; 3, 51–75%; and 4, 75–100%. To score tubular injury, the numbers of whole tubules per field were counted under ×400 magnification. The injury score was calculated in at least 10 randomly selected areas of the renal cortex, as follows: injury  score  (%) = (number  of  injured  tubules ÷ number of whole tubules) × 100.

### 2.5. Terminal Deoxynucleotidyl Transferase dUTP Nick End-Labeling (TUNEL) Assay

Apoptosis was semiquantitatively assessed using the TUNEL assay (Roche, Indianapolis, IN, USA). We counted the numbers of TUNEL-positive cells per field at 400x magnification and evaluated at least 10 random fields/slide. The mean number of brown cells was the number of TUNEL-positive cells. All counts were made by a single blinded observer using NIS-Elements BR 3.2 software (Nikon, Tokyo, Japan).

### 2.6. Immunohistochemistry

After deparaffinization, sections were incubated with primary antibodies against polyclonal anti-light chain 3B (LC3B; Cell Signaling Technology, Danvers, MA, USA), beta-galactosidase (*β*-gal), and 8-hydroxydeoxyguanosine (8-OHdG) (both from Abcam, Cambridge, MA, USA). Then, biotin-conjugated secondary IgG (1 : 200 dilution; Vector Laboratories, Burlingame, CA, USA), an avidin-biotin-peroxidase complex (Elite ABC Kit; Vector Laboratories), and DAB were added. We visualized sections under a light microscope and captured and analyzed digital images.

### 2.7. Protein Preparation and Western Blotting

Tissues were homogenized in lysis buffer and proteins (50 *μ*g) loaded and electroblotted. The blots were probed with polyclonal primary antibodies against Pink1 (Santa Cruz Biotechnology Inc., Santa Cruz, CA, USA); Parkin (Santa Cruz Biotechnology Inc.); p16INK4A and p62 (Abcam, Cambridge, UK); and LC3B, HMGB1, Mfn1, and Opa1 (Cell Signaling Technology) at 4°C overnight. The primary antibody was visualized by adding a secondary antibody and performing an electroluminescence assay (Amersham Pharmacia Biotech, Piscataway, NJ, USA).

### 2.8. Senescence-Associated *β*-Galactosidase (SA-*β*-Gal) Staining

To detect senescence, kidney tissues were fixed for 15 min in 1x fixative solution at room temperature, washed twice in PBS, and stained overnight at 37°C using the SA-*β*-gal staining kit (BioVision Inc., Milpitas, CA, USA) according to the manufacturer's instructions. The tissues were observed under a microscope; we sought development of a blue color. The sections were visualized under a light microscope and images were captured and digitally analyzed.

### 2.9. Cell Culture and Treatment

HK-2 human kidney proximal tubular cells (ATCC, Manassas, VA, USA) were cultured in renal epithelial basal medium (Gibco BRL, Grand Island, NY, USA) with manufacturer-provided supplements. Cells were incubated with 100 mg/mL ioversol or vehicle (PBS) for various times, with PBS or PC at various concentrations (0.2, 1, and 2 ng/mL), and then evaluated morphologically and via Western blotting. To verify the effect of PC on contrast-induced autophagy, five conditions were included: (1) untreated cells, (2) cells treated with ioversol (100 mg/mL), (3) cells treated with PC (1 and 2 ng/mL), (4) cells treated with chloroquine (CQ, 10 and 20 *μ*M), an inhibitor of autophagic flux, and (5) various combinations of these treatments. CQ was applied 4 h prior to the other agents. Cell lysate from each sample was loaded to ensure separation of LC3B-I and -II, and probed with LC3 antibody.

### 2.10. Mitophagy Detection

HK-2 cells were coloaded with 200 nM MitoTracker Green and 1 mM LysoTracker Red (Molecular Probes Inc., Eugene, OR, USA) for 20 min. Images were acquired using NIS-Elements BR 3.2 (Nikon). Mitophagy was determined by the double-positive cells of mitochondria with lysosomes. The numbers of double-positive cells were quantified.

### 2.11. Mitochondrial ROS Measurement

MitoSOX (Molecular Probes Inc.) was used to detect the mitochondrial ROS levels in HK-2 cells. Cells were incubated with 5 mM MitoSOX for 20 min and positive staining was subsequently detected by flow cytometry (FC500; Beckman Coulter, Indianapolis, IN, USA).

### 2.12. Statistical Analyses

Statistical analyses were performed using GraphPad Prism Software (ver. 8.0; GraphPad Software Inc., La Jolla, CA, USA). Data were evaluated using one-way ANOVA with Tukey's multiple comparison test (for comparison of all groups). In all statistical tests, *P* < 0.05 was taken to indicate significance. Values are presented as means ± standard errors of the means.

## 3. Results

### 3.1. Changes over Time in Renal Function and Pathology after Ioversol Administration

**Figure 1:**
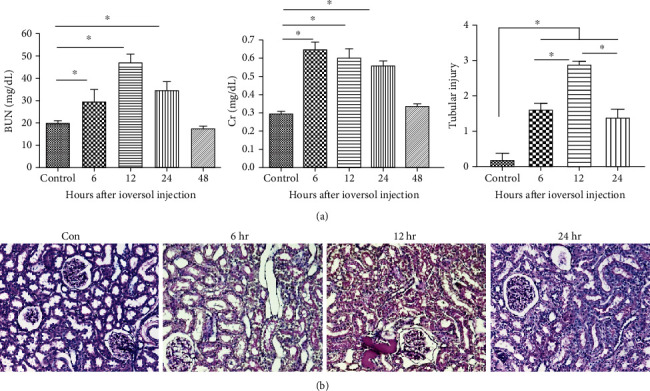
The contrast-induced renal dysfunction and histological injury in rats. (a) BUN and serum creatinine levels at 6, 12, 24, and 48 hours after contrast infusion. (b) Representative images of PAS staining in the tubular injury at 6, 12, and 24 hours after contrast infusion. Data were presented as mean ± SEM (^∗^*P* < 0.05).

We measured the levels of BUN and Cr 6, 12, 24, and 48 h after ioversol injection (Figure 1(a)). They were highest 6 and 12 h after contrast administration, respectively, and then decreased. Pathological examination revealed coarse, irregular vacuolization, cast formation, and loss of the brush border of renal tubular epithelial cells 6, 12, and 24 h after injection (Figure 1(b)). Thus, further studies were performed 12 h after injection.

### 3.2. Paricalcitol Attenuates Renal Dysfunction and Morphological Changes in CI-AKI

**Figure 2:**
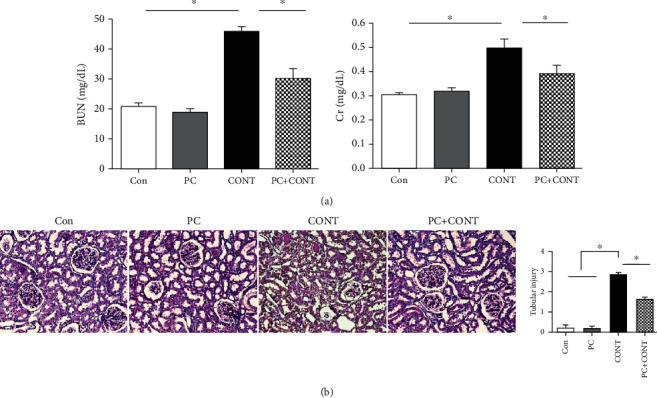
Effect of paricalcitol on biochemical tests and histological injuries in the kidney after ioversol administration. (a) Serum BUN and creatinine levels at 12 hours and (b) PAS staining in the kidney after ioversol administration. The injury score was determined as described in Materials and Methods. Images are representative of each group. Con: PBS-treated group; PC: paricalcitol-treated group; CONT: ioversol-treated group; PC+CONT: paricalcitol- and ioversol-treated group. Data were presented as mean ± SEM (^∗^*P* < 0.05).

The increases in BUN and Cr 12 h after ioversol injection were significantly decreased by paricalcitol pretreatment (Figure 2(a)). Severe tubular damage was evident after ioversol injection, and paricalcitol pretreatment inhibited such damage (Figure 2(b)).

### 3.3. Paricalcitol Reduces Contrast-Induced Apoptotic Cell Death and Oxidative Stress

**Figure 3:**
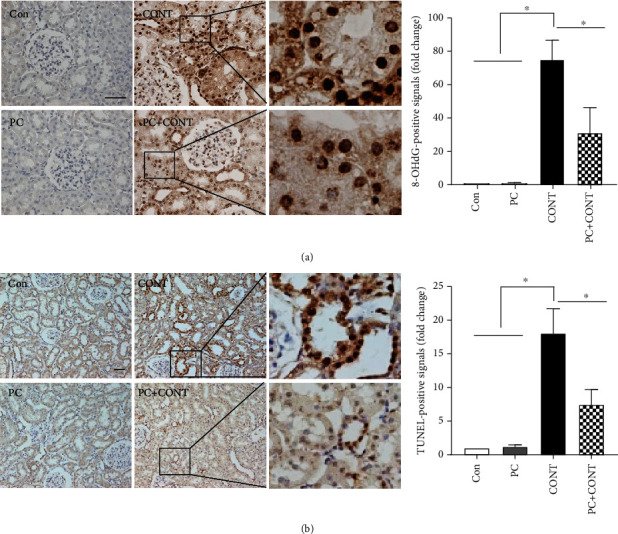
Effects of paricalcitol on oxidative stress and apoptosis in the kidney after ioversol administration. Immunohistochemical staining was performed with a specific antibody against 8-OHdG. Densitometric quantification for 8-OHdG was applied to each group. Ioversol-induced apoptosis was detected using the TUNEL assay. TUNEL-positive cells were stained with dense brown spots and counted as described in Materials and Methods. Apoptotic signals were found by TUNEL staining. Images are representative of each group. Con: PBS-treated group; PC: paricalcitol-treated group; CONT: ioversol-treated group; PC+CONT: paricalcitol- and ioversol-treated group. Data were presented as mean ± SEM (^∗^*P* < 0.05).

CI-AKI is associated with renal ischemia triggering oxidative stress [21, 24, 25]. Immunohistochemical staining for 8-OHdG, a ROS-induced marker of DNA damage, was used to investigate the effects of paricalcitol on CI-AKI. 8-OHdG-positive signals were evident in the nuclei of renal tubular epithelial cells exposed to ioversol, and paricalcitol pretreatment inhibited such signaling (Figure 3). We used TUNEL staining to investigate apoptosis. TUNEL-positive signals were found in the epithelial cells of dilated tubules, and their numbers increased 12 h after ioversol injection. Paricalcitol pretreatment inhibited the increase in numbers (Figure 3).

### 3.4. Paricalcitol Reduces the Renal Mitochondrial Damage Caused by Contrast

**Figure 4:**
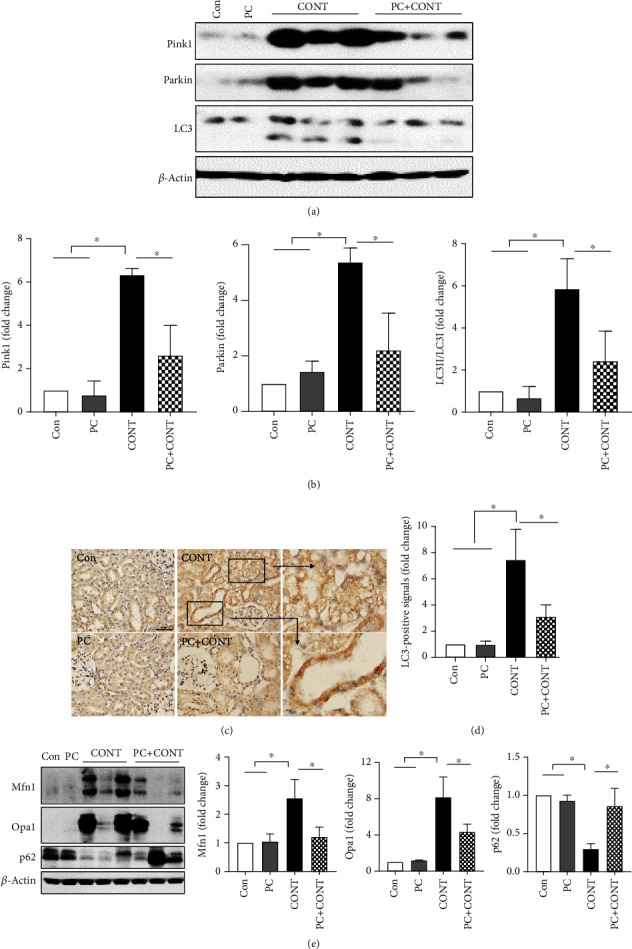
Effects of paricalcitol on mitophagy in the kidney after ioversol administration. (a, b) Immunoblot analysis was performed with a specific antibody against PINK1, Parkin, and LC3. *β*-Actin was used as loading control, and data were normalized against the density of *β*-actin. Blots are representative of each group. Cropped blots are displayed here, and full-length blots are included in the section of Supplementary Information. (c, d) Immunohistochemical staining was performed with a specific antibody against LC3. Densitometric quantification for LC3 was applied to each group. Images are representative of each group. (e) Immunoblot analysis for Mfn1 and Opa1 was performed to confirm the effect of paricalcitol on mitophagy, and p62 is for the effect of paricalcitol on the autophagic flux. Con: PBS-treated group; PC: paricalcitol-treated group; CONT: ioversol-treated group; PC+CONT: paricalcitol- and ioversol-treated group. Data were presented as mean ± SEM (^∗^*P* < 0.05).

Mitochondrial dysfunction and damage are involved in CI-AKI [5, 26]. Dysfunctional or damaged mitochondria are removed via mitophagy [26, 27]. Pink1- and Parkin-mediated mitochondrial autophagy is currently the best understood form of mitophagy in mammalian cells [28]. To examine whether paricalcitol protected against the renal mitochondrial damage caused by contrast, mitophagy-related factors were investigated. Ioversol administration induced kidney mitophagy, as reflected by increases in the expression levels of Pink1, Parkin, and LC3B-II (lower band) (Figures 4(a) and 4(b)). Paricalcitol pretreatment inhibited these increases (Figures 4(a) and 4(b)). In particular, LC3-positive tubular epithelial cells were observed in the kidney with ioversol injection, whereas their numbers were reduced by paricalcitol pretreatment (Figures 4(c) and 4(d)). To confirm the effect of paricalcitol on mitochondrial dysfunction caused by contrast, mitochondrial fusion proteins were examined; maintaining mitochondrial dynamics, including mitochondrial fusion and fission, is crucial for preserving mitochondrial function [29]. Levels of Mfn1 and Opa1, key regulators of mitochondrial fusion, were significantly increased only in the ioversol group, and pretreatment with paricalcitol attenuated their expression (Figure 4(e)).

### 3.5. Paricalcitol Decreases Contrast-Induced Senescence

**Figure 5:**
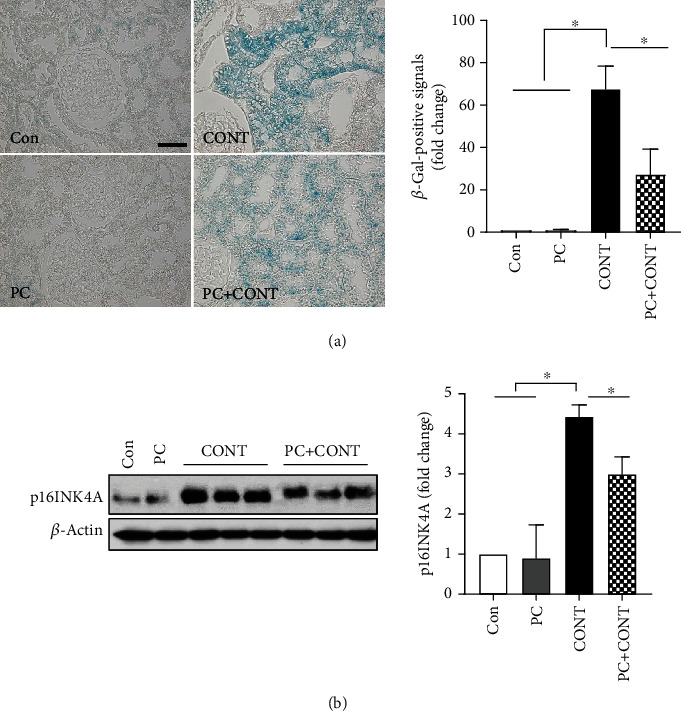
Effects of paricalcitol on senescence in the kidney after ioversol administration. (a) *β*-Galactosidase staining was performed with a specific assay kit. Densitometric quantification for *β*-galactosidase was applied to each group. Positive signals for *β*-galactosidase were observed as blue in the ioversol-treated kidney. Images are representative of each group. (b) Immunoblot analysis was performed with a specific antibody against p16INK4A. *β*-Actin was used as loading control, and data were normalized against the density of *β*-actin. Blots are representative of each group. Cropped blots are displayed here, and full-length blots are included in the section of Supplementary Information. Con: PBS-treated group; PC: paricalcitol-treated group; CONT: ioversol-treated group; PC+CONT: paricalcitol- and ioversol-treated group. Data were presented as mean ± SEM (^∗^*P* < 0.05).

Increased expression of senescence markers is evident in a variety of kidney diseases [30–32]. SA-*β*-gal is the most widely used biomarker of senescent and aging cells, being easy to detect both in situ and in vitro [33, 34]. SA-*β*-gal-positive signaling increased in renal tubules after ioversol administration, and PC inhibited this signaling (Figure 5(a)). The p16INK4A protein is another senescence biomarker [35, 36]. The expression level of renal p16INK4A increased in the ioversol group, and this increase was inhibited by paricalcitol (Figure 5(b)).

### 3.6. Paricalcitol Reduces Contrast-Induced Induced Mitophagy and Senescence in HK-2 Cells

**Figure 6:**
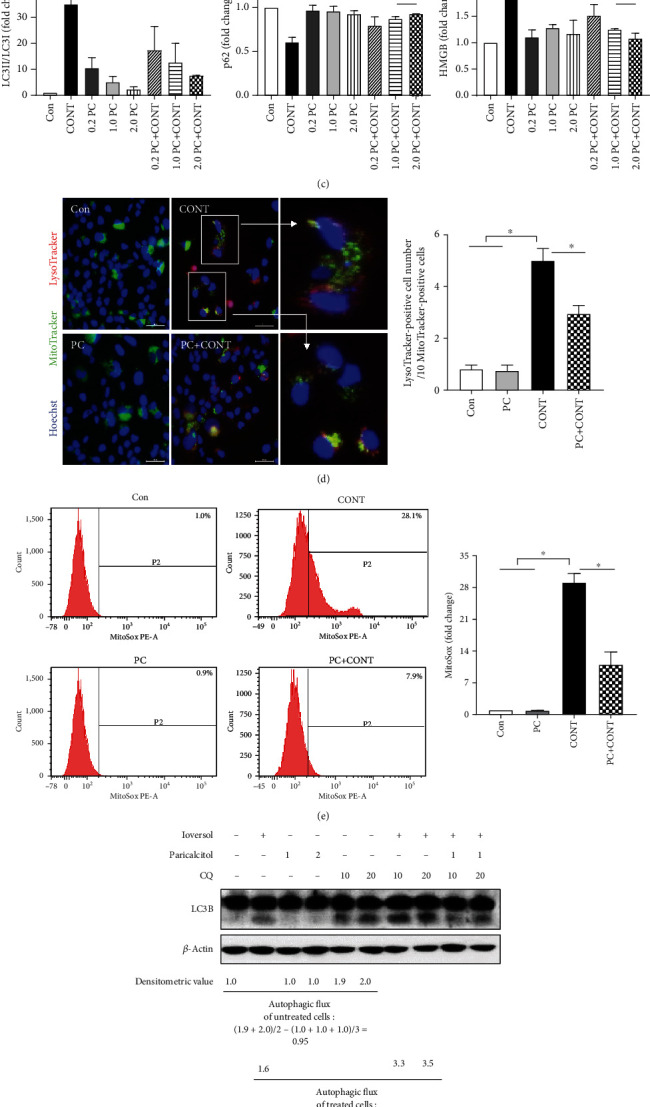
Effects of paricalcitol on contrast-induced cellular senescence in HK-2 cells. (a) Cell morphology was observed in each group at 60 min using phase-contrast microscopy under 200x magnification. HK-2 cells were treated with 100 mg/mL ioversol and 0.2, 1.0, and 2.0 ng/mL paricalcitol for 60 min. (b, c) Immunoblot analysis was performed with a specific antibody against LC3 and HMGB. *β*-Actin was used as loading control, and the ratio of LC3II/LC3I expression and the level of HMGB expression were analyzed by a densitometer. Blots are representative of each group. Cropped blots are displayed here, and full-length blots are included in the section of Supplementary Information. (d) Representative images of colocalization of lysosome (red—LysoTracker) and mitochondria (green—MitoTracker) in HK-2 cells. Mitophagy was detected by dual-positive staining. Hoechst (blue) was used for nuclear staining. (e) The mitochondria-associated ROS levels were measured by staining the cells with MitoSox, using flow cytometry. (f) Paricalcitol inhibited ioversol from inducing an autophagic flux. CQ is used as an inhibitor of an autophagic flux, and LC3B-II (lower band) expression is analyzed by Western blot. Con: no treatment; CONT: ioversol-treated group; PC+CONT: paricalcitol and ioversol-treated group. Data were presented as mean ± SEM (^∗^*P* < 0.05).

To confirm the protective effects of paricalcitol in terms of renal tubular damage, we explored whether paricalcitol would reduce ioversol-induced toxicity toward HK-2 cells. Paricalcitol did not affect cell morphology at any concentration tested (0.2, 1.0, and 2.0 PC in Figure 6(a)). Ioversol treatment alone (100 mg/mL) induced morphological changes (CONT in Figure 6(a)) that were initially evident 1 h after addition (data not shown). At this timepoint, paricalcitol-treated cells exhibited normal morphology (PC+CONT in Figure 6(a)). The high-mobility group box-1 (HMGB1) protein served as a marker of senescent cells [37]. Ioversol increased LC3B-II, p62, and HMGB1 expression, and paricalcitol significantly decreased expression at all concentrations tested (Figures 6(a)–6(c)). These results correlate well with the *in vivo* data (Figures 4 and 5). To examine whether paricalcitol protects against renal tubular cell injury caused by contrast, by reducing mitochondrial damage including mitophagy and mitochondrial oxidative stress, HK-2 cells were costained with green-fluorescing MitoTracker and red-fluorescing LysoTracker in the ioversol and ioversol with paricalcitol groups. Ioversol treatment increased the number of dual-positive cells, which was decreased by paricalcitol (Figure 6(d)). MitoSOX was employed to detect mitochondrial ROS in HK-2 cells. Paricalcitol significantly attenuated the increase in MitoSOX-positive cells caused by ioversol (Figure 6(e)). In addition, to determine whether autophagic flux is an induction or blockade effect exerted by ioversol, and whether paricalcitol can promote the control of autophagic flux, chloroquine (CQ), an autophagic flux inhibitor, was employed in HK-2 cells. As shown below, there was no LC3B-II (14 kDa) expression in the no-treatment and paricalcitol alone (1 and 2 ng/mL) groups. Treatment of HK-2 cells with ioversol led to LC3B-II accumulation, which was markedly increased by CQ. The effect of CQ appeared to be stronger in ioversol-treated cells (i.e., LC3B-II accumulation by ioversol was further enhanced by CQ), indicating increased LC3B-II turnover and induction of autophagic flux by ioversol. The enhanced LC3B-II accumulation induced by CQ and ioversol was decreased by paricalcitol treatment (Figure 6(f)).

## 4. Discussion

We found that ioversol compromised renal function in rats by triggering histopathological changes in, and apoptosis of, renal tubules as well as high expression levels of 8-OHdG, mitophagy and autophagy markers (Pink1, Parkin, Mfn1, Opa1, LC3, and p62), and senescence markers (*β*-gal and p16INK4A). Paricalcitol prevented renal dysfunction and attenuated renal damage by reducing oxidative stress and mitochondrial damage, and also reduced senescence *in vivo*. It also maintained HK-2 cell viability and decreased the expression levels of LC3-II and HMGB1 *in vitro*. In addition, an autophagy inhibitor was used to determine whether paricalcitol had an effect on autophagy or mitophagy. The autophagic flux itself did not change with paricalcitol treatment alone, but was induced by ioversol. This effect was inhibited by the autophagy inhibitor, and the LC3B-II accumulation was reduced by pretreatment with paricalcitol. These findings show that paricalcitol exerts its effects in CI-AKI by reducing the associated damage rather than increasing autophagy or mitophagy itself.

Oxidative stress is a key driver of CI-AKI [38]. Contrast augments kidney ROS formation and induces hypoxia. In our study, ioversol increased cellular oxidative stress and mitochondrial damage, and then autophagy and mitophagy were activated to protect against tissue injury. Some *in vivo* studies have shown that autophagy and mitophagy are both involved in the pathophysiology of radiocontrast-induced nephropathy (RCN) [26, 39, 40]. It has been suggested that, in the context of RCN, autophagy and mitophagy modulate apoptosis, oxidative stress, and inflammation. Autophagic removal of mitochondria is important for mitochondrial quality control. Poor-quality mitochondria may enhance cellular oxidative stress, generate apoptosis signals, and induce cell death [41]. In our study, mitochondrial ROS generation, damage, and mitophagy, which were increased by ioversol, were reversed by paricalcitol pretreatment. It was recently reported that stanniocalcin-1 (STC1) and tetramethylpyrazine (TMP) have protective effects on CI-AKI by regulating mitochondrial quality control and modulating tubular cell mitophagy, respectively [29, 42]. Exogenous recombinant human STC1 administration reduced the expression of mfn2, TOMM20, and p62 induced by iohexol. TMP reduced autophagosomes and the degree of mitophagy induced by iodine contrast, and prevented mitochondrial fragmentation by reversing the alterations in Drp1 and Mfn2 expression in an RCN rat model. The above two studies were similar to ours in terms of preventing RCN by regulating mitophagy via the administration of certain substances.

Autophagy is a term used to describe lysosomal-mediated degradation of proteins, lipids, and organelles, whereas mitophagy is defined as mitochondria-specific autophagy [41]. These two processes might seem to occur separately or simultaneously in our experiments. Due to oxidative stress induced by a radiocontrast agent, specific proteins in tubules might be become unfolded, abnormally modified, or mistargeted. These proteins are accumulated into tubular cells leading to cellular damages and finally cell deaths. Autophagy might be activated through these processes. On the one hand, ROS or reactive nitrogen species caused by oxidative stress might damage the mitochodria, its protein, DNA, and lipids resulting in a decrease in mitochondrial membrane potential or an increase in mitochondrial fission, and these have been shown to signal mitophagy. These changes stabilized Pink1 leading to the recruitment of Parkin to mitochondria resulting in mitophagy progression [43].

Yang et al. suggested that rapamycin has a renoprotective effect against CI-AKI, triggering high-level mitophagy that attenuates mitochondrial injury and oxidative stress [8]. Similarly, in the present study, mitophagy played a pivotal role in attenuating renal injury in our CI-AKI model. However, the processes in play may differ. Rapamycin has been found to be beneficial in an RCN model, enhancing mitophagy and thus attenuating serum levels of creatine and increasing ROS formation and apoptosis. On the other hand, in our study, paricalcitol attenuated renal injury by decreasing oxidative stress and apoptosis and reducing mitophagy through decreased mitochondrial damage. Additionally, we demonstrated that chloroquine, an autophagy inhibitor, attenuated the beneficial effects of paricalcitol in an *in vitro* experiment. These results show that mitophagy plays a critical role in pathophysiology of CI-AKI.

Cells exposed to stress signals can undergo apoptosis or enter into senescence (irreversible cell cycle arrest). The path taken depends on the cell type and the nature of the stress [44–46]. In AKI models, the absence of p16INK4A, a senescence marker, promotes regenerative cell proliferation and improves outcomes [36, 47, 48]. In biopsy specimens from patients with kidney injuries and in experimental animals, induction of senescence (reflected by increases in p21CIP1, p16INK4A, and SA-*β*-gal levels) increases disease progression [30, 49, 50]. The senescent cell burden in the kidney may usefully predict prognosis. In our study, p16INK4A overexpression was evident within 6 h after contrast infusion (data not shown), peaked at 12 h (Figure 5(b)), and the peak was sustained for 24 h (data not shown), similar to the SA-*β*-gal expression pattern. It is thus likely that AKI caused by contrast is associated with early renal tubular epithelial cell cycle arrest, triggering sustained senescence. Previous studies have shown that paricalcitol exhibits antioxidative and anti-inflammatory effects [51, 52]. We found that the extent of DNA oxidation (as determined by 8-OHdG levels) was increased by contrast and attenuated by paricalcitol. Therefore, paricalcitol might minimize the development of senescent cells and/or reduce the total senescent cell burden of AKI.

The association between autophagy and senescence is complex, being heavily dependent on the cell types involved and the nature of the stress [53]. Autophagy may play dual roles in the regulation of cellular senescence, either inducing or inhibiting the process [54–56]. Autophagy may normally prevent senescence by eliminating potentially dangerous elements. However, when the damage overloads the autophagic capacity, senescence is activated and autophagy contributes to such activation under stressful conditions [57]. Consistent with these suggestions, we found that the levels of both autophagy and senescence markers increased in our CI-AKI model, and these changes were attenuated by paricalcitol pretreatment.

## 5. Conclusions

Our findings suggest that mitophagy- and senescence-promoting pathways may be activated in a rat CI-AKI model, and that paricalcitol exerts a renoprotective effect by reducing damage to mitochondria and senescent cells.

## Data Availability

The data used to support the findings of this study are included within the article.

## Supplementary Materials

Supplementary Materials Experimental schematic diagram.
