# Supplementary material for: Paricalcitol Attenuates Contrast-Induced Acute Kidney Injury by Regulating Mitophagy and Senescence
Source: Oxid Med Cell Longev. 2020 Nov 23;2020:7627934. doi: 10.1155/2020/7627934 (PMC7704155; doi:10.1155/2020/7627934)
Supplement: Supplementary Materials — Experimental schematic diagram. [file 7627934.f1.docx]

**Supplemental Figure**

**Sparague Dawley rat.** Male. 10 wk

**Paricalcitol :** 0.3 ug/kg, i.p.

**Contrast : Ioversol,** 8.3ml/kg, tail vein

**Indomethacin :** 10mg/kg, tail vein

**L-NAME :** 10mg/kg, tail vein

**Paricalcitol**

**Indomethacin**

**L-NAME**

**Ioversol**

15 min

30 min

**Paricalcitol**

30 min

24 hr

6 hr

6 hr

12 hr

**Sac.**

**Sac.**

**Sac.**
